# The Crystal Structure of *Toxoplasma gondii* Pyruvate Kinase 1

**DOI:** 10.1371/journal.pone.0012736

**Published:** 2010-09-14

**Authors:** Rebecca Bakszt, Amy Wernimont, Abdellah Allali-Hassani, Man Wai Mok, Tanya Hills, Raymond Hui, Juan C. Pizarro

**Affiliations:** 1 The Structural Genomics Consortium (SGC), University of Toronto, Toronto, Ontario, Canada; 2 Molecular Biotechnology, Department of Physics and Measurement Technology, Biology and Chemistry (IFM), Linköping University, Linköping, Sweden; University of Cambridge, United Kingdom

## Abstract

**Background:**

Pyruvate kinase (PK), which catalyzes the final step in glycolysis converting phosphoenolpyruvate to pyruvate, is a central metabolic regulator in most organisms. Consequently PK represents an attractive therapeutic target in cancer and human pathogens, like Apicomplexans. The phylum Aplicomplexa, a group of exclusively parasitic organisms, includes the genera *Plasmodium*, *Cryptosporidium* and *Toxoplasma*, the etiological agents of malaria, cryptosporidiosis and toxoplasmosis respectively. *Toxoplasma gondii* infection causes a mild illness and is a very common infection affecting nearly one third of the world's population.

**Methodology/Principal Findings:**

We have determined the crystal structure of the PK1 enzyme from *T. gondii*, with the B domain in the open and closed conformations. We have also characterized its enzymatic activity and confirmed glucose-6-phosphate as its allosteric activator. This is the first description of a PK enzyme in a closed inactive conformation without any bound substrate. Comparison of the two tetrameric *Tg*PK1 structures indicates a reorientation of the monomers with a concomitant change in the buried surface among adjacent monomers. The change in the buried surface was associated with significant B domain movements in one of the interacting monomers.

**Conclusions:**

We hypothesize that a loop in the interface between the A and B domains plays an important role linking the position of the B domain to the buried surface among monomers through two α-helices. The proposed model links the catalytic cycle of the enzyme with its domain movements and highlights the contribution of the interface between adjacent subunits. In addition, an unusual ordered conformation was observed in one of the allosteric binding domains and it is related to a specific apicomplexan insertion. The sequence and structural particularity would explain the atypical activation by a mono-phosphorylated sugar. The sum of peculiarities raises this enzyme as an emerging target for drug discovery.

**Enhanced version:**

**This article can also be viewed as an enhanced version in which the text of the article is integrated with interactive 3D representations and animated transitions. Please note that a web plugin is required to access this enhanced functionality. Instructions for the installation and use of the web plugin are available in Text S1.**

## Introduction

During glycolysis, pyruvate kinase (PK) catalyzes the irreversible phosphorylation of ADP at the expense of phosphoenolpyruvate (PEP), yielding pyruvate and ATP. PK also regulates glycolytic flux, serving as a switch between the glycolytic and the gluconeogenic pathways, and the supply of glycolytic phosphometabolites used as synthetic precursors in cellular proliferation [1].

Allostery is a critical mechanism of regulating PK activity. The most common form of allosteric regulation for PK is its upregulation by fructose-1,6-bisphosphate (FBP), which increases the affinity and reduces the cooperativity of substrate binding [2]. However, other sugars have also been shown to regulate PK activity; for example, fructose 2,6-bisphosphate is the primary allosteric effector in trypanosomatids [3]. In mammalian developing tissues, the M2 isoform of PK is expressed and then replaced in differentiated cells by an alternatively spliced variant, M1-isozyme, that is not allosterically regulated. Two additional allosterically regulable isozymes, PK L/R, are encoded by another gene with alternative promoters to produce the liver form (L) or the erythrocyte form (R) [4].

Several observations suggest that allosteric regulation of pyruvate kinase is a critical feature in proliferating cells and tissues. First, tumors often lose expression of M1 and regain expression of the PK-M2 [5], [6], which is advantageous to the unique metabolism of tumor cells because its regulation by both fructose-1,6-bisphosphate and tyrosine phosphorylated proteins gives tumors the metabolic flexibility to shuttle glucose byproducts into anabolic versus catabolic processes, according to its growth requirements [1], [7],[8]. Second, microorganisms that exhibit obligate or facultative anaerobic growth, including bacteria and yeast, possess allosterically regulated PK enzymes [9], [10]. Third, a variety of parasitic protozoans, including trypanosomatids and apicomplexans, express allosterically regulable forms of pyruvate kinase which allow them to adapt their glycolytic flux to the different cell environments encountered throughout their life cycle progression [3], [11], [12], [13].

*Toxoplasma gondii* is an obligate intracellular parasite of warm-blooded animals including humans, and belongs to the phylum *Apicomplexa*, which includes other medically relevant organisms such as *Plasmodium falciparum* and *Cryptosporidium parvum*, the causative agents of malaria and cryptosporidiosis, respectively. *T. gondii* infection causes toxoplasmosis, a significant health issue for pregnant women and immunocompromised individuals [14]. The life cycle of this parasite initiates when its invasive forms are released into the host gut, where they invade host cells to reproduce. Carbohydrates appear to be the main source of energy for the rapidly multiplying forms of *Toxoplasma*, and the glycolytic pathways provide the main catabolic route to consume them [15]. *T. gondii* possesses two genes coding for pyruvate kinases, which have very distinct biochemical and enzymatic properties. PK1 is considered to be a cytoplasmic enzyme involved mainly in the glycolysis pathway, while PK2 localizes to the apicoplast and is postulated to be involved in fatty acid synthesis [16]. PK1 encoded by *T. gondii* appears to be the key regulator of the glycolysis pathway since neither hexokinase nor phosphofructokinase enzymes are allosterically regulated [15].

Previous characterization of cytoplasmic *T. gondii* pyruvate kinase (*Tg*PK1), using either parasite extracts [15] or a recombinant purified enzyme [13], indicated that this enzyme was allosterically activated by glucose 6-phosphate (G6P), which enhanced its activity under PEP unsaturated conditions. Since G6P is produced in the initial step of glycolysis by a hexokinase-catalyzed reaction, this would imply a different regulatory mechanism of the glycolytic flux in *Toxoplasma* parasites when compared to its mammalian hosts, which are typically allosterically regulated by a metabolite produced further down the pathway, namely FBP. This observation also underscores that glycolytic enzymes, despite their ubiquity, are shaped by evolution to best serve the organism's lifestyle.

A structural understanding of the peculiarities of *Tg*PK1 in relation to its enzymatic function would increase *Tg*PK1's value as a chemotherapy target, as has been established for other parasites, e. g. trypanosomatids [17]. To this end, we solved the structure of *Tg*PK1 in two configurations that provide insight into its allosteric regulation, and have compared its enzymatic properties with other apicomplexan PK enzymes.

## Results

### Enzymatic characterization and allosteric regulation

We have cloned and over-expressed the *Tg*PK1 as a recombinant protein in *E. coli*. The purified enzyme was found to be active and a tetramer in solution based on its elution profile on size exclusion chromatography (data not shown). The enzyme was characterized enzymatically, with apparent *K_m_*, *V_max_* and Hill's coefficient (*n*_H_) values obtained for PEP (Table 1). The values here reported are in agreement with those previously reported for this enzyme [13].

**Table 1:** Enzyme kinetic parameters of *Tg*PK1 determined in presence or absence of phosphorylated sugars.

	*K_m_*	*V_max_*	*n_H_*
	(PEP mM)	(mM NADH/min)	
No sugar	0.75±0.051	0.042±0.0012	1.71±0.17
G6P	0.12±0.008	0.044±0.0008	1.45±0.11
G1P	0.47±0.057	0.029±0.0014	1.61±0.28
F6P	0.75±0.104	0.044±0.0024	1.41±0.22
F1P	0.58±0.081	0.028±0.0016	1.56±0.30
FBP	0.55±0.044	0.055±0.0018	1.69±0.19
RBP	0.50±0.049	0.038±0.0014	1.67±0.23

Apparent *K_m_*, *V_max_* and Hill coefficient (n_H_) values are reported plus or minus their associated errors.

**Figure 1:**
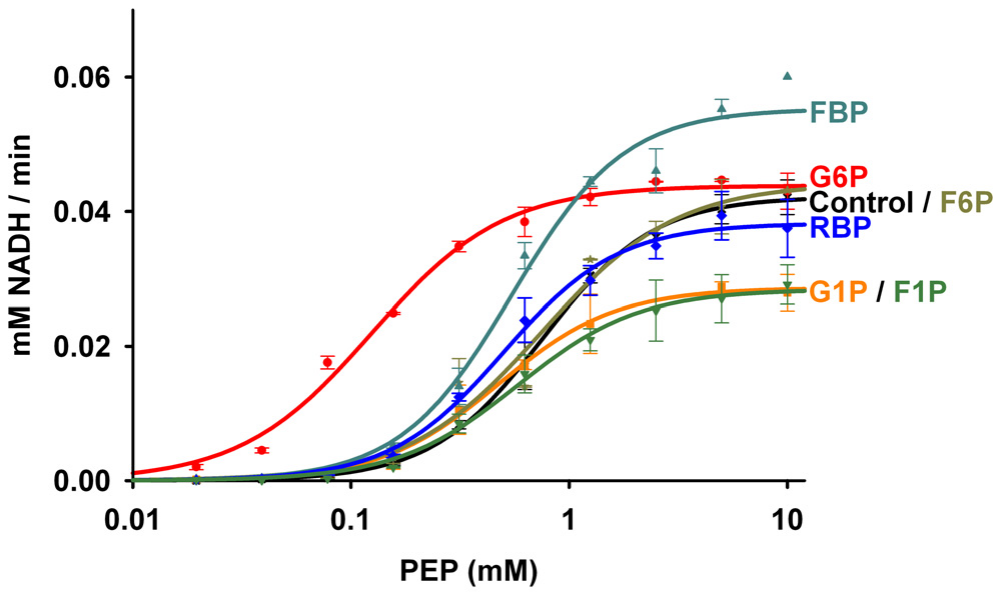
*Tg*PK1 enzymatic activity in the presence of mono and biphosphorylated sugars. Graph of velocity against concentration of Phosphoenolpyruvate. Derived kinetic constants are presented in Table 1.

The effect of various phosphorylated sugars on the enzymatic activity of *Tg*PK1 was also tested (Table 1 and Fig. 1). Although the *T. gondii* enzyme showed activation by FBP and G6P, each sugar affected the kinetic parameters differently. While G6P showed a classic allosteric activation with a six-fold reduction in the apparent *K_m_* and no effect on the *V_max_*. FBP did not impact significantly the Michaelis constant but increased the *V_max_* by 20%. Both G1P and F1P behaved as allosteric inhibitors with a 40% reduction in the *V_max_*. The two remaining phosphorylated sugars tested, F6P and RBP, showed no significant effect on the *Tg*PK1 enzymatic.

### TgPK1 crystallization and overall structure description

We solved the structure of two different constructs of *Tg*PK1: the full-length enzyme (Met1 to Glu531 plus its N-terminal 6×His tag), which crystallized in the absence of substrates, effectors or added ions (“full *Tg*PK1”); and an N-terminal truncated version starting at position Ile39 (“truncated *Tg*PK1”). Crystals of the truncated *Tg*PK1 were grown in the presence of K^+^, Mg^2+^ and a potential inhibitor, although no evidence for this compound was found in the electron density. The full length crystal lacked electron density for the initial 40 residues, the same ones absent in the truncated form. Equivalent residues have been removed from human PK-R with no effect on its enzymatic activity [18]. Therefore the differences observed between these two structures are most likely not related to the N-terminal truncation but rather the result of different crystallization conditions.

**Figure 2:**
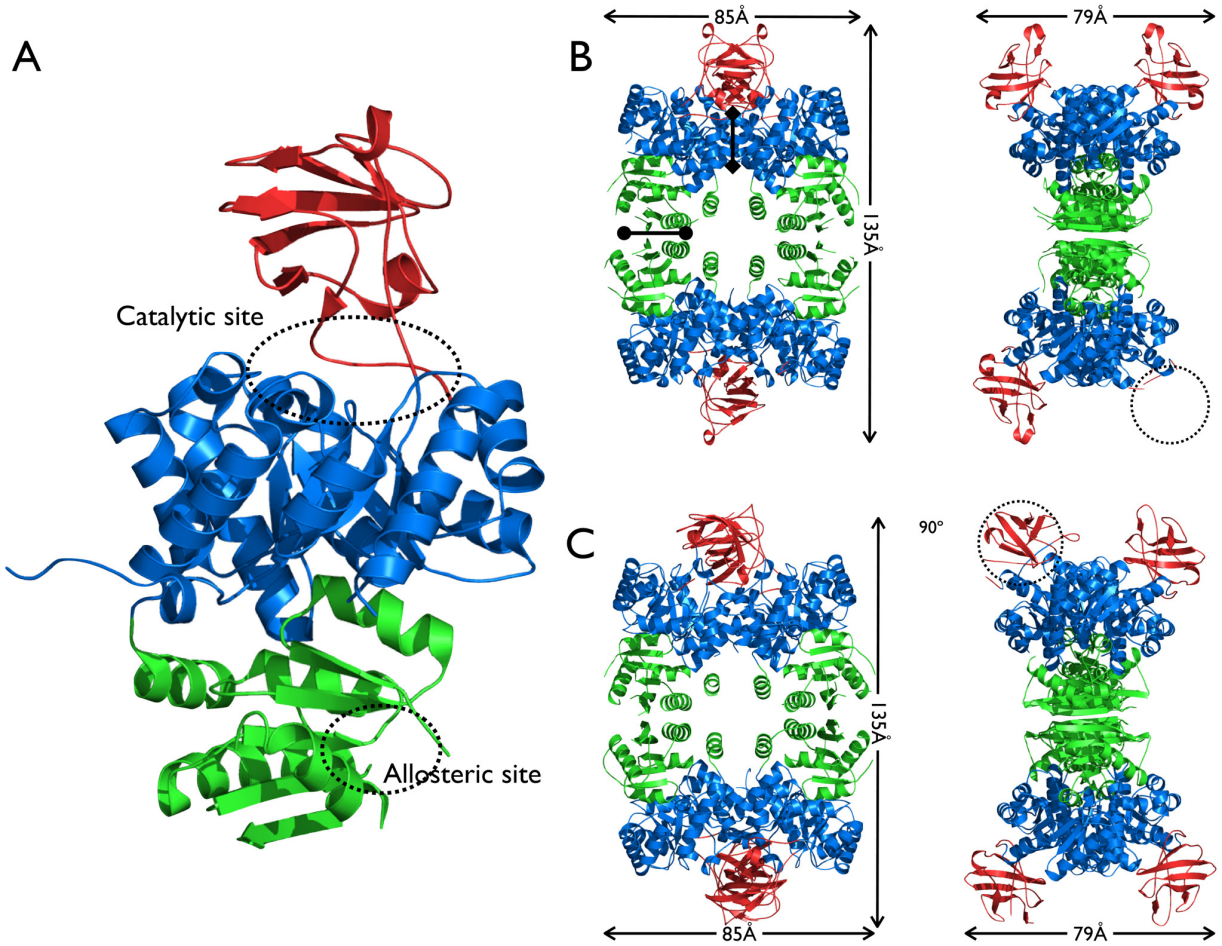
Structure of *T. gondii* pyruvate kinase. A) Monomeric structure. The A, B, and C domains are colored blue, red and green respectively. The catalytic site at the interface of the A and B domains, and the allosteric site in domain C, are highlighted. B and C) Orthogonal views of the homotetrameric organization of full *Tg*PK1 (B) and truncated *Tg*PK1 (C). The tetramer interface A–A′ is indicated by a line with diamond shaped ends, and the C–C′ by a line with filled circles at its ends. Dotted circles indicate a disordered B domain in the full structure; and a “closed” conformation in the truncated structure.

The quaternary structure of the *Tg*PK1, in both the full and the truncated structures, is a tetramer resembling previously described pyruvate kinase structures, e.g. *E. coli* PK (1PKY, rmsd 1.2 Å over aligned Cα atoms and 47% sequence identity) and *S. cerevisiae* PK (1A3X, rmsd 1.3 Å over aligned Cα atoms and 43% sequence identity). In both crystals, the asymmetric unit is composed of a tetramer with imperfect D2 symmetry. Each molecule in the tetramer is connected through their A and C domains to other units. The two A–A′ interfaces in the tetramer bury 1557 and 1691 Å^2^ in the full and 1774 and 2021 Å^2^ in the truncated form and the C–C′ excluded from solvent on average 620 Å^2^ and 720 Å^2^ in the full and truncated forms respectively. This asymmetry creates a “dimer-of-dimers” oligomeric structure. Despite the differences in the areas of contact, both tetramers showed the same overall dimensions, 135×85×79 Å (Fig. 2 and supplementary Datapack S1). However, the full and truncated *Tg*PK1 tetramers did not show the same relative orientation between the different monomers. Therefore, the superposition of one chain does not result in a perfect match across the whole oligomeric structure.

Each monomer is composed of four domains: A, B, C and N [19], [20]. The central A domain (spanning residues Ile59-Gly124 and Val224-Cys393) is composed of an (α/β)_8_ barrel. The B-domain (from Pro125 to Pro223) is composed of only β-strands and random coils. The catalytic site is located at the interface of these two domains, where residues in domain A interact with PEP and ADP and residues from the B domain contact ADP and Mg^2+^ [21]. The C domain extends from residues Val394 to Glu531 and is composed of α and β structural elements. This domain contains the effector binding site (also called the allosteric site) [22], [23]. Finally, an N-terminal domain includes the first fifty amino acids of the protein and is a helix-loop-helix motif, however in the *Tg*PK1 only a single helix is observed (Fig. 2A).

**Figure 3:**
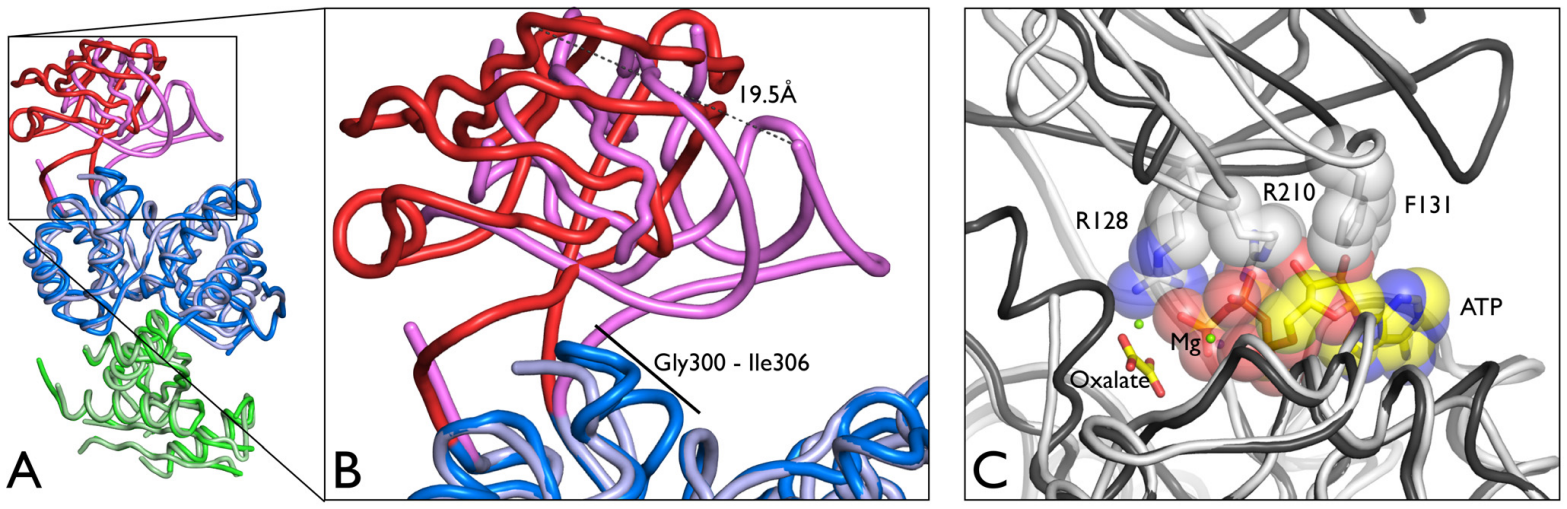
The B domain exists in multiple conformations. A) Superposition between full (Chain B) and truncated (Chain B) *Tg*PK1 structures, in dark and lighter tones, respectively. B) The detail shows two views of the B domain movement in the open (red) versus closed (pink) conformations. C) Superposition between the A domains of the active rabbit PK-M1 in complex with ATP and oxalate (1A49[21]) with closed short *Tg*PK1 (darker and lighter tones respectively). Side chains from the *Toxoplasma* enzyme clashing with the ligands are represented.

The monomers from the full length *Tg*PK1 structure were all similar to each other, superposing over all atoms with rmsd values under 1 Å (Table 2). A notable difference among the four monomers was the complete absence of electron density for the B domain in one of the chains (D), which has not been reported for any PK structure (Fig. 2B). Within the crystal lattice this domain was exposed to a solvent channel and the closest crystallographic neighbor is a C domain from chain C. The missing domain had little to no influence over the quality of the A domain within chain D, whose b-factors were similar to the average of the structure. Conversely, in the truncated *Tg*PK1 structure all of the B domains were visible in the electron density, with one monomer remarkably displaying a closed conformation for this domain (Fig. 2C). This large structural rearrangement involving a rigid body motion of the B domain resulted in a maximum displacement of nearly 20 Å (Fig. 3 and supplementary Datapack S1). This conformational change mimics the one that takes place during the normal catalytic cycle of the pyruvate kinase when the binding of PEP and ADP triggers the closure of the cleft between domains A and B. In the truncated *Tg*PK1 structure this closed conformation was observed despite an empty active site. Steric hindrance effects would prevent the binding of the nucleotide to this conformation; consequently we defined it as inactive. We used the program DynDom [24] to characterize this domain movement. Two hinge bending motion regions were identified containing two conserved Pro residues, P125 and P216, at which point the B domain pivots towards the A domain by rotating ∼68°, generating a 77% closed conformation compared to the open conformation. Repeating the same analysis to compare open conformations of the two *Tg*PK1 structures among them showed a range of rotation angles between 3.4° and 7.4°, with the closing percentage ranging from 13 to 89%. This compares with rotations of 20° to 26° and closures of 88 to 98% when inactive (T) and active (R) conformations have been analyzed for the mammalian PK-M1 and M2 [25].

**Table 2:** All atoms superposition of the four monomers of the *T. gondii* pyruvate kinase 1.

rmsd (Å)	A	B	C	D
A	–	0.841	0.8	0.886
B	5.19	–	0.759	0.985
C	1.214	5.336	–	1.003
D	1.193	5.256	0.971	–

rmsd values are presented above the diagonal for the full form and below the diagonal for the truncated construct.

**Figure 4:**
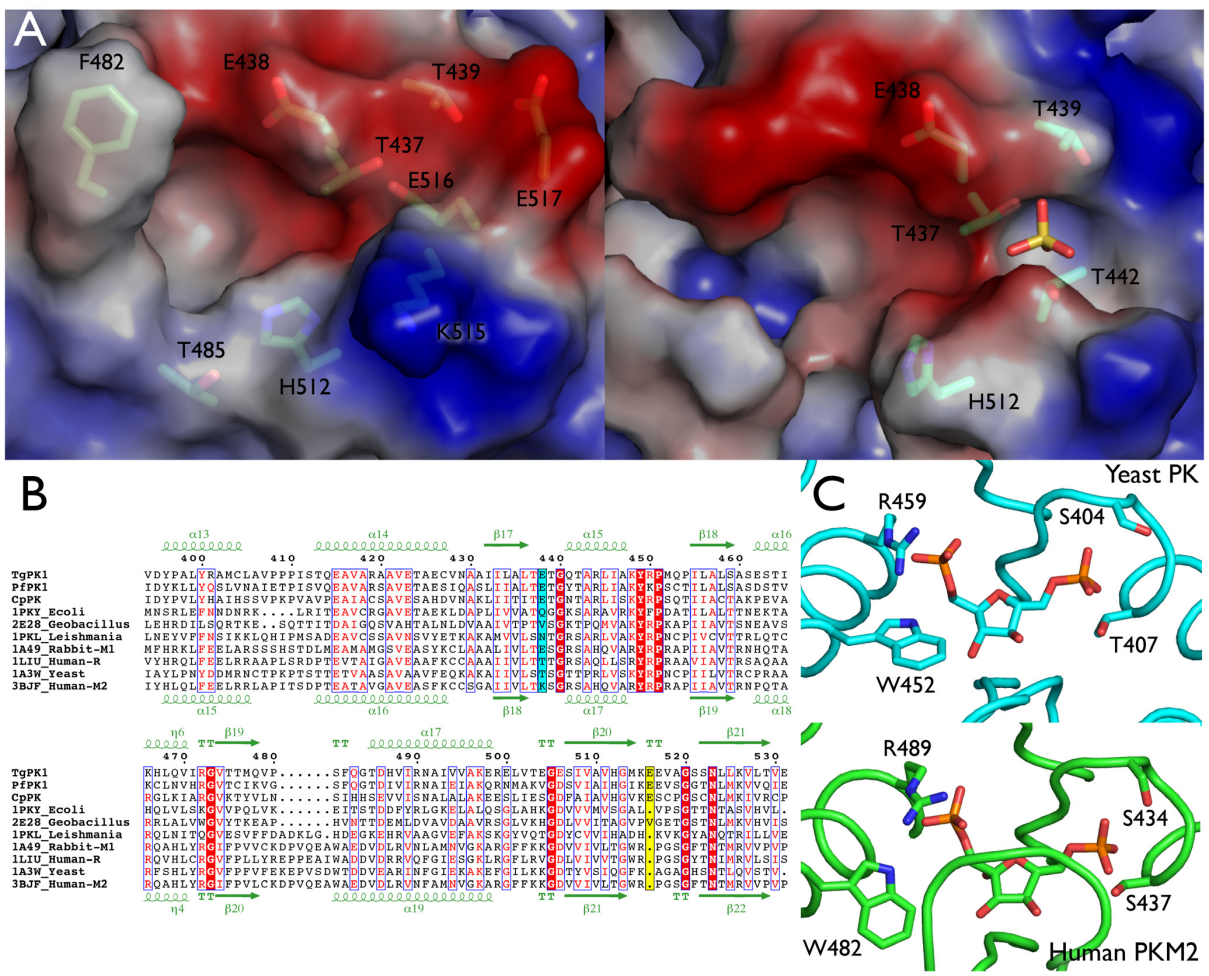
Allosteric site. A) The effector site of the two observed conformations in the *Tg*PK1 structures colored according to surface electrostatic potential. Left: empty site (ordered conformation); Right: a sulfate molecule occupies the effector site. B) Multiple sequence alignment color coded according to sequence similarity of pyruvate kinases from *T. gondii* (TgPK1), *P. falciparm* (PfPK1), *C. parvum* (CpPK), *E. coli*, *Geobacillus stearothermophilus*, *Leishmania mexicana*, Rabbit PK-M1, human PK-R, Yeast (*S. cerevisiae*) and human PK-M2. Positions 438 and 516 are highlighted in cyan and yellow respectively. The top and bottom lines indicate the elements of secondary structure of the *T. gondii* and human PK-M2 enzymes respectively. C. FBP binding sites of the yeast PK (1A3W[23]) and human PK-M2 (3BJF[8]). Side chains that interact with the phosphate moieties are indicated.

**Figure 5:**
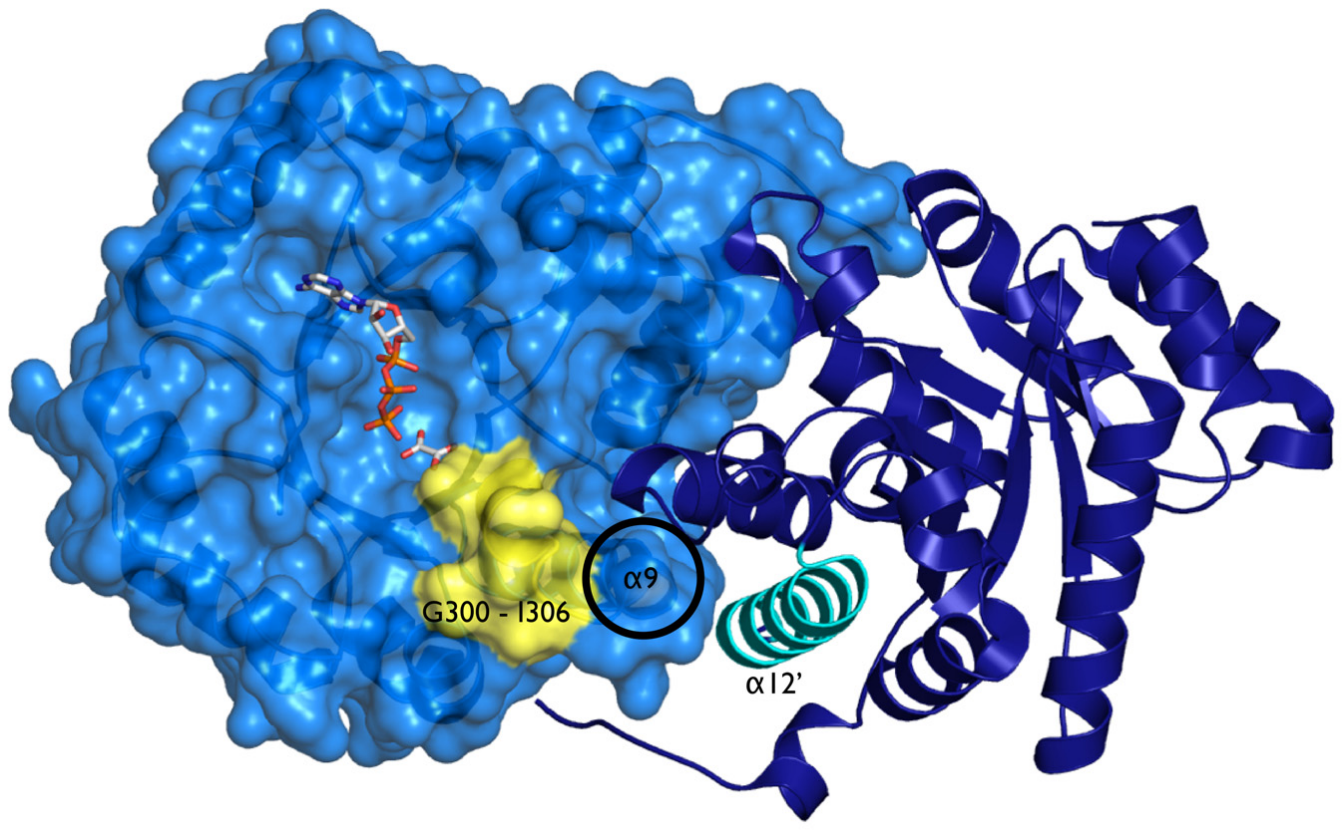
The A–A′ interface of *Tg*PK1. The A domain is represented as a transparent surface and the A′ domain as a cartoon representation. The position of the Gly300-Ile306 loop is indicated in yellow. Helix α9 from the A domain is indicated with a circle, and its interacting helix in the A′ domain, α12 is colored in cyan. ATP and oxalate are shown for reference, with their positions derived from a superposition with 1A49[21]. The scene is viewed through the B domain (but both B domains have been excluded for clarity).

The full and truncated *Tg*PK1 data were predominantly well-ordered and the final models covered most of the sequence with the exception of the previously described B domain and the initial 40 N-terminal residues in the full length structure. Additional disordered regions included several loops in the full length C domain (Val407-Pro409, Pro480-Thr485 and Lys515-Ser522). All these regions are solvent exposed and with the exception of the last one they are not involved in the C–C′ tetramer interaction surface. It is noteworthy that the Pro480-Thr485 loop (corresponding to the mobile loop in yeast PK [23]) and Lys515-Ser522 regions are part of the allosteric binding site. In the case of the truncated *Tg*PK1, the Lys515-Ser522 loop showed two conformations: an ordered conformation where the electron density for two Glu residues, 516 and 517, was well defined, and a disordered conformation lacking electron density for the region between residues 515 and 522 (Fig. 4A and supplementary Datapack S1). In the disordered conformation, additional electron density was observed and modeled as a sulfate molecule binding three side chain hydroxyl groups of residues Thr437, Thr439 and Thr442. Finally, a disordered region was exclusively associated with the closing of the AB cleft in the truncated *Tg*PK1, the Gly300 to Ile306 loop (Fig. 3 and 5). Interestingly, this loop was ordered despite the absence of a single conformation for the B domain in Chain D of the full length structure.

## Discussion

### Structural flexibility and enzymatic activity

Prior studies have highlighted the important effect of PK's structural flexibility on its catalytic activity, particularly the movement of the B domain [21], [26]. The open conformation of the B domain is an inactive state (Tight). Upon binding of both substrates, it closes through a hinge motion to the active conformation (Relaxed), which allows for the transfer of the phosphate group from PEP to ADP [21]. However the two *Tg*PK1 structures reported here have revealed an extreme flexibility extending the repertoire already present in the ∼30 PK structures available in the Protein Data Bank. First, the full length structure showed three monomers in an open inactive conformation and one subunit where there was no electron density for the B domain (disorder likely due to lack of crystal contacts). This is consistent with the B domain existing in solution as a continuum of open conformations. Second, the truncated *Tg*PK1 also showed two different conformations for the B domain, a closed inactive conformation in one of the subunits, and an open inactive conformation in the three remaining subunits. The larger rotation of the *Tg*PK1 B domain rendered this conformation inactive. A superposition with an active conformation of the pyruvate kinase, rabbit PKM2 in complex with oxalate and ATP [21], highlights potential steric hindrances between *Tg*PK1 B domain residues – Arg128, Phe131 and Arg210 – and the phosphonucleotide. This closed inactive conformation illustrates the dynamic nature the *Toxoplasma* enzyme and expands the range of movements of the pyruvate kinase B domain.

Pyruvate kinases show some degree of cooperativity between the subunits implying a structural linkage between them [27]. How this is achieved is not well understood, but structural reports on the same enzyme under different crystallographic conditions [25], in complex with different ligands [21], or from mutants modifying the inter-subunit communications [28], have shown subtle but measurable changes in the tetramer shape, either in translation or rotation of the different domains. In our case, the translation component was very limited since both structures showed similar dimensions of the homotetramer, within 1 Å (Fig. 2 and supplementary Datapack S1). However, the quaternary structures are clearly different, since the buried surfaces involved in the tetramer assembly differ by more than 10%. These differences can be explained by small rotations (1 or 2°) along the A–A′ and the C–C′ interfaces, that create some ∼2 Å displacements. In both of our *Tg*PK1 structures, one of the B domains showed a conformation outside of the average open inactive conformation present in the other three subunits, which was associated with a change in the dimerization surface interface along the A domains but not the C domains. In the full length structure the differences between the A–A′ dimer interfaces are smaller when compared to the truncated *Tg*PK1 but in both cases the smaller buried surface is associated with the outlier B domain conformation. These slight structural differences have a profound impact on the enzyme kinetics and are responsible for the regulation of the enzyme activity.

How the movement of B domain impacts the dimer interface is poorly understood [2], [22], [29]. In the *T. gondii* PK1 structure, changes in the B domain orientation are linked to displacements in several regions in the A domain, but one in particular, the Gly300 to Ile306 loop located at the double intersection of the A and B domains and the A–A′ dimer interface, stands out (Fig. 5 and supplementary Datapack S1). This region contains a conserved residue Glu305 whose side chain wedges between the A and B domains, making a hydrogen bond (average distance in the full length structure ∼2.9 Å and ∼3.0 Å in the truncated) with the Nδ2 of Asn214, a neighboring residue of the hinge region. In the closed conformation of domain B, the whole Gly300-Ile306 loop is disordered and the position of the Asn214 Cα shifts over 3 Å in the two conformations. Our hypothesis links the order-disorder transition of this loop with the closing of the B domain. The Gly300-Ile306 loop is connected to helix α9, therefore the B-domain movement could be relayed to the A–A′ interface via this helical element (Fig. 5). In support of this, the changes in the A–A′ surface observed in the truncated *Tg*PK1 structure involved several residues located in helices α9 and also α12. The α12 is a partner of helix α9 in the A′ domain and the last secondary structure element of this domain directly linked to the C domain, the allosteric binding domain. One might be inclined to suggest that a conformational change in the C domain induced upon binding of an allosteric regulator could then affect the adjacent subunit. Changes in the relative position of these two helices or enhanced structural flexibility could facilitate the movement of the B domain and consequently increase affinity for the substrate. These changes observed in the different subunits of the *Tg*PK1 could be explained by other mechanisms but the proposed model addresses two observations – the cooperativity among subunits and the linking between the allosteric site and the catalytic cleft.

### Allosteric regulation

The *T. gondii* PK1 enzyme is allosterically activated by glucose-6-phosphate. This was unexpected since this enzyme contains a glutamic acid at position 438, which has been considered a hallmark of unregulated enzymes like mammalian PK-M1 [13] (Fig. 4B). It is generally thought that a positively charged amino acid is required to bind the allosteric effector FBP, but little is known about the binding requirements for other allosteric compounds. In the case of human PK-M2, the Nε atom of the Lys side chain coordinates the 1′-phosphate moiety of FBP. However, the yeast PK binds FBP and possesses a Thr residue at that position; and *Leishmania mexicana* PK, that is also regulated by a biphosphorylated sugar (fructose-2,6-biphosphate), has an Asp residue at this position (Fig. 4B). Therefore, having a bulky, negatively charged residue like Glu at this position would be expected to hinder the binding of positively charge entities like biphosphorylated sugars but not monophosphorylated sugars. In the truncated *Tg*PK1, two monomers have a sulfate group in the region Thr437-Thr442. The presence of a sulfate group in the allosteric binding had been reported for other structures and had been postulated to occupy the position allocated for the phosphate moiety of the effectors [10], [25], [30]. This region is involved in the binding of the 6′ phosphate group of FBP in human PK enzymes, M2 and R, and in the yeast PK. The largest stereochemical hindrance for the binding of biphosphorylated effectors in *Tg*PK1 is His512, a residue situated at the bottom of the allosteric binding site. This residue is conserved among the parasitic enzymes and, in its current rotamer, would shift any allosteric compound position with respect to the FBP as recognized in either the PKM2 or the yeast structures (Fig. 4C).

Unexpectedly, we observed an ordered conformation in the allosteric binding site with well-defined electron density for two regions (Pro480-Thr485 and Lys515-Ser522), absent in previously determined structures of PK without effectors [22], [23]. In the new conformation present in the truncated *Tg*PK1, when the Lys515-Ser522 is ordered, the carboxylate group of Glu516 occupies the position of the sulfate group (Fig. 4A). This residue appears to be specific to the apicomplexan PKs and is absent in both the bacterial and in other eukaryotic pyruvate kinases (Fig. 4B). Additionally, in this conformation the Pro480 to Thr485 loop was well defined. This section is associated with the binding of the 1′phosphate from FBP in the PKM2 and the yeast PKs. However, these enzymes possess a six-residue insertion when compared to the apicomplexans, creating a larger helical element (Fig. 4B) that forms one of the walls of the effector-binding site. It contains a tryptophan whose indole ring nitrogen interacts with the 1′phosphate group together with the side chain from an arginine (Fig. 4C). A smaller loop is also observed in *Geobacillus stearothermophilus* PK an enzyme that is regulated by a monophosphorylated sugar, (ribose-5-phosphate) but also in the *E. coli* type I PK enzyme that is affected by FBP [2], [10]. The short loop Pro480-Thr485 creates a more limited allosteric site. Overall, the charge environment seen in the *Tg*PK1 structure is more neutral when compared to the human PKM2 and the yeast PK, consistent with the binding of mainly monophosphorylated sugars. In both the PKM2 and the yeast PK, a transition was observed between the effector free structure and the bound form; in both cases an ordering occurred upon binding of the FBP [22], [23]. In the case of the *T. gondii* enzyme, the allosteric site was ordered in the absence of any effectors or sulfates. Furthermore, it appears that Glu516 carboxyl group could mask the binding site of the phosphate moiety. Taken together, these differences in sequence and three-dimensional structure lead us to propose that the binding mode of G6P would be significantly different from the yeast and the mammalian enzymes binding mode of FBP.

In summary, we have solved the structures of two different forms of the *T. gondii* pyruvate kinase 1 that reveal an unprecedented degree of flexibility in the B domain. We hypothesize that the B domain flexibility is linked to the dynamic nature of the PK structure in general and that the *Tg*PK1 structures reported here highlight this aspect. The closed conformation challenges our structural understanding of the PK catalytic cycle, since it is assumed that the closure of the active site cleft requires the binding of both substrates, PEP and ADP. The initial N-terminal residues, which are absent in the truncated *Tg*PK1 may influence the B domain conformation. Despite this caveat, the structure illustrates the conformational changes that accompany the closure of the B domain. These new structures of the PK enzyme have allowed us to propose a mechanism that rationalizes the enzymatic characteristics of the *T. gondii* pyruvate kinase, one that could be generalized to other apicomplexan PKs. In addition, the elucidation of the parasitic enzyme structure is relevant to the development of chemical entities or probes by means of virtual and enzymatic screening. Specific inhibitors or activators of the *Tg*PK1 would be extremely useful reagents to explore the function of the enzyme *in vivo* (chemical genetics) and will represent the validation steps for a drug development program against *T. gondii*.

## Materials and Methods

### Cloning and protein production

The full-length synthetic template of 55.m00007 (ToxoDB, http://www.toxodb.org/) was ordered from Codon Devices (Cambridge, MA, USA) and the expression constructs were subcloned from it. Specifically, full length *Tg*PK1 (Met1 to Glu531) and the N-terminally truncated protein (Ile39 to Glu531, henceforth referred to as truncated *Tg*PK1) were cloned with a N-terminal His6-tag followed by a TEV protease cleavage site. Proteins were expressed as previously described [31]. Briefly, clones were grown in TB media in a LEX bioreactor system (Harbinger Biotechnology and Engineering Corp., Markham, Ontario, Canada). Overnight starter cultures were left to grow at 37°C until reaching an OD_600_ value around 5, cooled to 15°C, and subsequently induced with 0.5 mM IPTG overnight at 15°C. Cells were harvested by centrifugation and the pellets re-suspended in 40 ml per liter of culture in binding buffer (50 mM HEPES pH 7.5, 500 mM NaCl, 5 mM imidazole, 5% glycerol) with protease inhibitors, 1 mM benzamidine and 1 mM phenylmethyl sulfonyl fluoride (PMSF), then flash frozen in liquid nitrogen and stored in −80°C until needed. Resuspended pellets were pretreated with 0.5% CHAPS and 500 U of benzonase for 40 minutes at room temperature and cells were mechanically lysed with a microfluidizer (Microfluidizer Processor, M-110EH). The cell lysate was centrifuged to eliminate cells debris and the cleared lysate was loaded onto a DE52 (Whatman, MA, USA) anion exchange resin followed by a 2.5 mL Ni-NTA (Qiagen, MD, USA). The Ni-NTA column was then washed with 200 mL of a buffer consisting of 50 mM HEPES pH 7.5, 500 mM NaCl, 30 mM imidazole and 5% glycerol. The protein was eluted with 15 mL of a buffer made of 50 mM HEPES pH 7.5, 500 mM NaCl, 250 mM imidazole and 5% glycerol. The eluted sample was further purified by size exclusion chromatography in a Superdex 200 26/60 (GE Healthcare, NJ, USA) column equilibrated with a buffer consisting of 10 mM HEPES, pH 7.5 and 500 mM NaCl. The peak fractions eluting at 164 mL for the full length and 168 mL for the truncated *Tg*PK1 (consistent with a tetrameric enzyme for both forms) were pooled and the protein identity was evaluated by SDS-PAGE and mass spectroscopy. This purified protein was used for enzymatic characterization and crystallography without removal of the N-terminal purification 6×His tag in either case.

### Enzyme characterization

Kinetics experiments were carried out at room temperature using a lactate dehydrogenase-coupled spectrophotometric assay in a Synergy 2 microplate reader (BioTek, Vermont, USA)[32]. The kinetic parameters for PEP were determined by modifying its concentration, from 0.01 to 10 mM, in the following reaction mixture, 0.2 mM NADH, 2 mM ADP, 50 mM HEPES pH 7.5, 50 mM KCl, 20 mM MgCl_2_, 10 U of rabbit muscle lactate dehydrogenase type II (Roche), and 1 µg/ml of the full length *Tg*PK1. The same method was used to test the effect of several sugars over the enzyme activity, including, fructose-1-phosphate (F1P), fructose-6-phosphate (F6P), glucose-1-phosphate (G1P), glucose-6-phosphate (G6P), fructose-1,6-biphosphate (FBP) and ribulose-1,5-biphosphate (RBP). All sugars were used at a fixed concentration of 1 mM. Reactions were monitored for 6 minutes and kinetic parameters were obtained by fitting initial rate against substrate concentration by a nonlinear curve-fitting algorithm (SigmaPlot 2000 software; SPSS Inc., Chicago, USA).

### Crystallization, data collection and structure determination

Purified full length *T. gondii* PK1 was crystallized using the sitting drop vapor diffusion method. Crystals for the full length structure were obtained by mixing one part of a protein solution at 10 mg/ml (10 mM HEPES pH 7.5 500 mM NaCl) with one part of reservoir solution containing, 19% PEG3350 100 mM BisTris pH 6.0 and 200 mM NH_4_I. Crystals appeared in less than a week. Crystals were flash cooled in liquid nitrogen and cryo-protected in mother liquor supplemented with 15% glycerol. The initial diffraction pattern showed high mosaicity and resolution to ∼3.0 Å. The crystal was subjected to an annealing procedure by plunging in Paratone-N oil then re-freezing in a cryo-stream, which significantly improved the mosaicity and the resolution limit. Diffraction data were collected at the X25 beamline at NSLS (National Synchrotron Light Source at Brookhaven National Laboratory, NY, USA). The diffraction data were integrated and scaled using the HKL2000 software package [33]. The structure was solved by molecular replacement using a full monomer structure from yeast pyruvate kinase (PDB id. 1A3X) as the search model and the program Phaser [34]. Crystals of the truncated *Tg*PK1 were also grown by sitting-drop vapor diffusion technique over a reservoir of 15% PEG3350 and 100 mM succinic acid pH 7.0. One part of the reservoir solution was mixed with one part protein solution, truncated *Tg*PK1 at 10 mg/ml in 10 mM Hepes pH 7.5, 50 mM NaCl, 150 mM KCl and 5 mM Mg_2_SO_4_. Crystals were cryoprotected by supplementing mother liquor with 15% ethylene glycol prior to flash cooling at −170°C. Diffraction data were collected at our in house X-ray facility, equipped with an X-ray generator FR-E SuperBright (Rigaku, TX, USA) and an imaging plate detector R-AXIS HTC (Rigaku, TX, USA), at 2.2 Å. The phases of the truncated *Tg*PK1 structure were obtained by molecular replacement using the full length structure as a search model. For both structures, model building was performed with COOT [35] and the structures were refined with REFMAC5 [36] from the CCP4 suite of programs [37]. The stereochemistry of the both models was checked by MOLPROBITY [38]. Relevant data collection and refinements statistics are shown in Table 3. The coordinates for the structures and their structure factors have been deposited with the Protein Data Bank (PDB accession codes, 3EOE for the full length and 3GG8 for the truncated *Tg*PK1). Structural superpositions were performed by LSQKAB [39] as implemented in CCP4; tetramer interfaces were calculated with the PISA server [40] (http://www.ebi.ac.uk/msd-srv/prot_int/pistart.html); domain movements were analyzed using the DynDom server [24] (http://www.sys.uea.ac.uk/dyndom/); images of sequence alignments were prepared using ESPript/ENDscript [41]; and structure figures were generated with Pymol (DeLano Scientific, Palo Alto, California, USA. http://www.pymol.org).

**Table 3:** Summary of crystal parameters and refinement statistics.

	full *Tg*PK1	truncated *Tg*PK1
Space group	P2_1_	P2_1_
a (Å)	98.04	109.02
b (Å)	130.68	92.31
c (Å)	113.85	112.44
β	117.4°	105.57°
Resolution range (Å)	50–2.3 (2.4–2.3)	50–2.2 (2.28–2.2)
Unique reflections	103408 (5921)	105806 (10323)
Redundancy	3.6 (2.3)	3.8 (3.8)
R_merge_ (%)	6.9 (58.8)	8.8 (91.3)
Completeness (%)	93.2 (53.8)	97.9 (96.3)
I/σ(I)	12.1 (1.2)	11.4 (2.0)
PDB code	3EOE	3GG8
R-value	0.20 (0.29)	0.20 (0.28)
R_free_	0.25 (0.33)	0.26 (0.35)
r.m.s bonds (Å)	0.008	0.011
r.m.s angles (°)	1.118	1.282
Number of atoms		
Protein	13383	14291
Organic	–	15
Solvent	657	490
Ramachandran plot		
Favored	1743/1774	1831/1885
Allowed regions	29/1774	52/1885
Outliers	2/1774	2/1885
Average B factors (Å^2^)	36.3	35.3

## Supporting Information

Datapack S1 Standalone iSee datapack - contains the enhanced version of this article for use offline. This file can be opened using free software available for download at http://www.molsoft.com/icm_browser.html.
(ICB)

Text S1 Instructions for installation and use of the required web plugin (to access the online enhanced version of this article).
(PDF)
